# Evaluation of Boride Layers on C70W2 Steel Using a New Approach to Characterization of Boride Layers

**DOI:** 10.3390/ma15113891

**Published:** 2022-05-30

**Authors:** Andrijana Milinović, Josip Stojšić, Ivica Kladarić, Božidar Matijević

**Affiliations:** 1 Mechanical Engineering Faculty in Slavonski Brod, University of Slavonski Brod, 35000 Slavonski Brod, Croatia; jstojsic@unisb.hr (J.S.); ikladaric@unisb.hr (I.K.); 2 Faculty of Mechanical Engineering and Naval Architecture, University of Zagreb, 10000 Zagreb, Croatia; bozidar.matijevic@fsb.hr

**Keywords:** boride layer, morphology, volume fraction of the boride phase, microstructure, microhardness

## Abstract

In this study, boride layers on C70W2 steel, obtained by boronizing at temperatures ranging from 870 to 970 °C and durations from 4 to 8 h, were investigated. The characterization of the layers was carried out using a new approach based on the change in the volume fraction of the boride phase. Analysis of the change in volume fraction showed that an increase in temperature and duration resulted in thicker layers, with temperature having a greater influence. Based on the volume fraction of the boride phase, the layer is divided into compact and toothed parts. With increasing temperature, the thicknesses of both parts of the layer increased. The thickness of the toothed part was the highest after 6 h of boronizing and further prolongation of boronizing led to a decrease in the thickness. Regression equations were estimated for the prediction of the volume fraction of the boride phase, the thickness of the compact part, and that of the toothed part of the boride layer as a function of the boronizing parameters. This study has shown that characterization based on the volume fraction of the boride phase offers advantages over the conventional method based on the average thickness of the layer.

## 1. Introduction

Boronizing is a thermochemical process in which boron atoms diffuse from the boronizing agent into the surface of the workpiece. Upon penetration into the surface, boron atoms bond with the atoms of the base material, forming single-phase or dual-phase intermetallic compounds (borides) on the surface. The resulting surface layers are characterized by exceptional hardness, which gives boronized surfaces excellent resistance to abrasion wear at room temperature and elevated temperatures. Furthermore, boronizing increases the resistance to adhesion wear, oxidation, short-term corrosion resistance in acidic environments, and resistance against corrosion in molten metals [1,2,3,4].

Boronizing can be applied to a wide range of ferrous and non-ferrous materials [5,6]. The thickness and quality of the surface layers obtained depend on the boronizing parameters (temperature, duration, available quantity of active boron in the boriding agent) and the chemical composition of the substrate [1,7,8,9]. Boronizing can be carried out in solid (powder or paste), liquid, and gaseous mediums. Although it has drawbacks, such as long process durations, treatment cost, and difficulty in controlling process variables, pack boronizing is still most frequently used [5,7,10,11,12,13].

The boronizing of steels is usually performed at temperatures between 800 and 1050 °C with treatment durations between 2 and 16 h. The surface layers that form may consist of one (Fe_2_B) or two (FeB/Fe_2_B) phases. Although the FeB phase is harder than Fe_2_B, the formation of a layer consisting of only the Fe_2_B phase is preferred because of the brittleness of FeB. In addition, FeB and Fe_2_B have different coefficients of thermal expansion, which can lead to the formation of microcracks at their interface, which can cause FeB to spall under high loads [7,14,15,16,17,18].

It can be said that all thermodiffusion layers are mainly characterized by their thickness and hardness [11]. Most surface treatments result in layers that are parallel to the surface, so determining their average thickness is not problematic. In some processes (i.e., cementation and nitriding), the effective thickness of the surface layer is defined by the depth at which the achieved hardness value is specified in the relevant standards. Although boronizing has been in use for many years, there is still no standard that clearly prescribes the method of determining the average thickness of the boride layer. The problem of the lack of a unique methodology for determining the thickness of the boride layer is further complicated by its unusual morphology. Namely, boronizing results in a surface layer of characteristic saw-tooth morphology that can be more or less pronounced. Toothness is more pronounced in boronized non-alloyed and low-carbon steels, while the boronizing of alloyed and high-carbon steels reduces the degree of toothness [1,3,7,8,19,20]. The determination of the average layer thickness is crucial for many studies of the boronizing process. Many authors reported the problem of determining the average layer thickness in their studies and stated that the definition of the layer thickness is not obvious due to its saw-tooth morphology [1,4,5,15,21,22]. 

**Figure 1:**
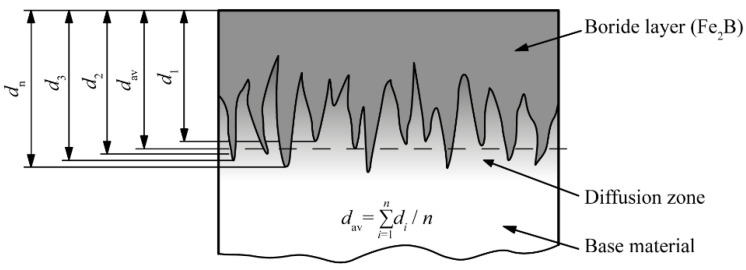
Determining the average thickness of the boride layer.

Since there is no standard, different authors define the average layer thickness in different ways. Most authors define the average layer thickness as the arithmetic mean of the distance from the surface to the top of the boride teeth [5,9,11,12,22,23,24]. This method is shown in Figure 1. Panda et al. measured the thickness as the distance from the surface of the sample to the approximate middle of the Fe_2_B teeth [25].

As Yu et al. pointed out, this approach to determining the average layer thickness does not take into account the width of the tooth [15], so some authors determine the average layer thickness as the ratio of the area of the boride layer and the total length [7,15]. 

The lack of a uniform standard, on the one hand, and the different methods used to determine the average layer thickness, on the other hand, make it difficult to compare research results. Matijević found in his study that when determining the layer thickness using different methods (metallographic line method, planimetry method, and image analyzer), there is significant scatter in the results of up to 35% [26]. Karakaş et al., when comparing the values of activation energy obtained in their research with the values obtained in other studies, stated different approaches to determining the average layer thickness as the first of the possible causes of the differences [7]. 

It can be said that for better characterization of boride layers, data on the average thickness are not sufficient, as they do not express anything about the morphology, i.e., the toothness of the layer. In terms of quality, boride layers with pronounced toothness are preferable, as they ensure good adhesion of the layer to the surface [3,10,17,27]. Although many authors emphasize the importance of toothness, there are no papers quantifying the interdependence of layer morphology and boronizing parameters. It is similar to tribological tests in which the relationship between the test results and layer morphology is mostly expressed qualitatively, with layers being described as more or less toothed [17,27]. Since the morphology of the layer is extremely important for its quality, it is necessary to quantify it. Depending on the morphology, the boride layer can be divided into the compact part (the part with a large share of the boride phase) and the toothed part (the remaining part of the boride layer). From the tribological point of view, it can be said that the compact part of the layer is the primary carrier of wear resistance. On the other hand, the toothed part provides good adhesion of the layer to the substrate.

Taking into account all the mentioned disadvantages of the usual methods for the characterization of boride layers, a new approach to additional characterization is proposed in this study, based on the analysis of the volume fraction of the boride phase in the cross-section. It can be said that this paper has two objectives. One objective is to investigate the influence of boronizing parameters (temperature and duration) on the morphology and properties of boride layers on C70W2 steel. The second objective is to verify the applicability of the proposed characterization method and its comparison with common characterization methods based on determining the average boride layer thickness as described in the introductory part. The investigation is carried out on C70W2 steel, which belongs to the group of unalloyed tool steels. As one of the steels with the lowest carbon content within this group, characterized by good toughness, it is used for the production of forging hammers and tools, punches for paper and leather, tools for soft stone, and tools for engraving and woodworking [28]. One of the main disadvantages of this steel, its low abrasion resistance, could be greatly improved by boronizing. In this way, the application of this steel could be extended to the production of tools for processing harder materials and for parts exposed to oxidizing and erosive fluids (cutting tools for soft metals, paper and leather, oil burner and pressure steam nozzles, conveyor rollers, baffles and swirl elements for fluids with or without solid particles, etc.).

## 2. Materials and Methods

The study of boride layers was carried out on C70W2 carbon steel (the chemical composition is given in Table 1).

Boronizing is performed according to a 3^2^ factorial design of experiment with 3 repetitions of each experiment, with temperatures of 870, 920, and 970 °C and durations of 4, 6, and 8 h. According to the chosen experimental plan, 27 specimens with dimensions of Ø 16 × 7 mm were made. The specimens were cleaned and sanded with 600-grit emery paper before boronizing. Boronizing was performed in Hef-Durferrit Durborid 3 powder (solid medium for boronizing at temperatures between 850 and 1000 °C) in an LHP laboratory furnace (manufactured by ES, Samobor, Croatia) without a protective atmosphere. After boronizing, the specimens were cut into cross-sections and prepared for metallographic examinations (mounted in cold mounting acrylic resin, ground with emery paper up to 1000 grit, polished with alumina, and etched with 3% nital). The microstructures were examined using optical microscopy (with the Leica DM 2500 M microscope and the Leica Q550 MW imaging solution).

**Figure 2:**
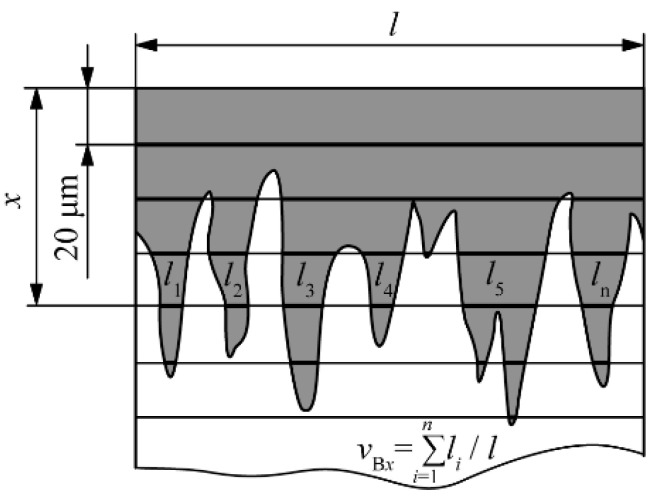
Determination of the volume fraction of boride phase.

The determination of the change in the volume fraction of the boride phase in the cross-section of the layer was carried out by the method of linear quantitative metallography, in which the volume fractions of the individual phases are calculated as the ratio of the sum of lengths of all the segments passing through these phases and the length of the test line (Figure 2). The analysis of the fraction of the boride phase in the cross-section was performed every 20 μm from the surface. Micrographs recorded at a magnification of 100:1 were used for the analysis, and digital image magnification was used for an easier and more accurate reading. 

After determining the change in the volume fraction of the boride phase with depth, the dependence of the layer morphology on the boronizing parameters was analyzed. Based on the volume fractions, the layer was divided into compact and toothed parts. To the best knowledge of the authors of this study, this approach to studying the morphology of the boride layer has not been reported in studies by other authors. Therefore, the authors decided that the depth at which the volume fraction of the boride phase was 95% should be considered the compact part of the layer. The remaining part of the layer represented the thickness of the toothed part of the layer.

The microhardness of the boride layers was measured using a Vickers microhardness tester at a test load of 0.981 N (HV0.1) and a dwell time of 15 s. Assuming that the hardness primarily depends on the proportion and hardness of each phase (in this case, boride and base material), it is possible to determine the value of hardness more accurately if the volume fractions at each depth are known. The cross-sectional microhardness of Fe_2_B and the base material was estimated every 20 μm from the surface. The change in surface hardness in the cross-section was determined based on the volume fraction of the boride phase and the base material and the measured hardness according to the expression

(1) $$H_{L}=v_{B}\cdot H_{B}+\left(1-v_{B}\right)\cdot H_{BM}$$

where *H_L_* is the total hardness of the layer at the observed depth/HV, *H_B_* is the hardness of the boride (Fe_2_B) phase/HV, *H_BM_* is the hardness of the base material at the observed depth/HV, *v_B_* is the volume fraction of the boride (Fe_2_B) phase at observed depth/%, and (1 − *v_B_*) is the volume fraction of the base material at the observed depth/%. 

The experimental results were also statistically analyzed using the analysis of variance (ANOVA), response surface methodology, and regression analysis. ANOVA was performed to determine whether the effect of each input variable on the output data was significant. The null hypothesis states that the input variables have no effect on the output at a certain level of significance. The alternative hypothesis states that there is an effect between input and output variables. One way to test the null hypothesis is to use the *F*-statistic and *p*-value [28]. For the test of statistical significance, a confidence level of 95% (alpha level of 0.05) was chosen, which corresponds to a *p*-value of 0.05. If the *p*-value is less than 0.05, the null hypothesis should be rejected and replaced with the alternative hypothesis. The response surface methodology is concerned with finding the surface in the set of all regression surfaces that provides the best fit to the measured data, i.e., finding the surface with the minimum sum of the squares of the distances between the measured points and that surface. The response surface methodology uses regression analysis, which is performed in many practical problems where it is necessary to draw a conclusion and establish the dependence between dependent (output) and independent (input) variables based on a series of measurements [29].

## 3. Results

### 3.1. Microstructure

**Figure 3:**
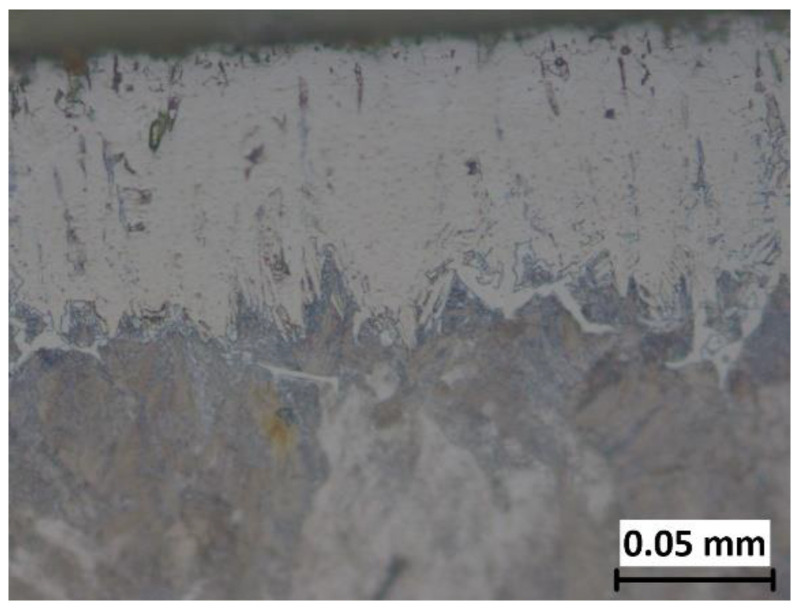
Optical micrograph of a layer boronized at 920 °C for 4 h, magnification 500:1.

The analysis of the microstructure showed that layers with less-pronounced toothness were obtained in all samples, which is commonly observed in boronized steels with a higher content of carbon or alloying elements. Figure 3 shows the microstructure of a layer boronized at a temperature of 920 °C for 4 h. The darker area just below the layer is the result of a higher share of carbides in the diffusion zone.

**Figure 4:**
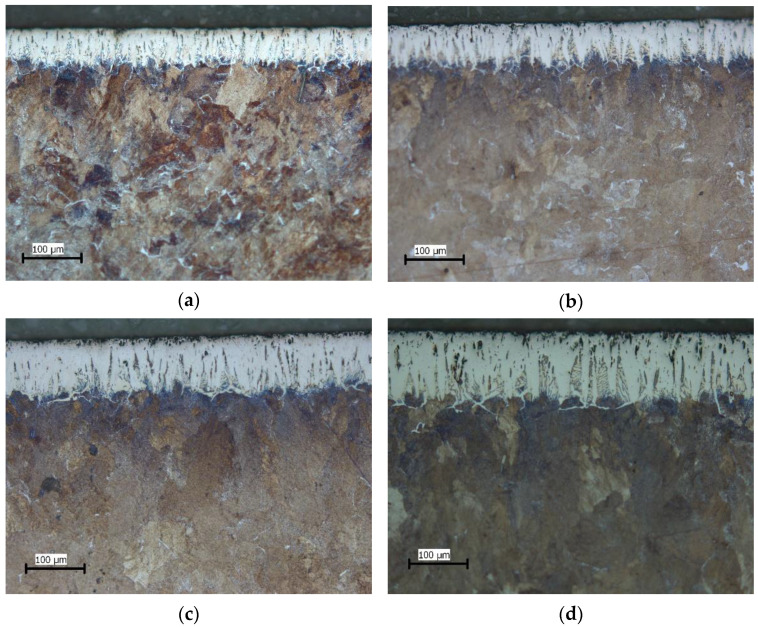

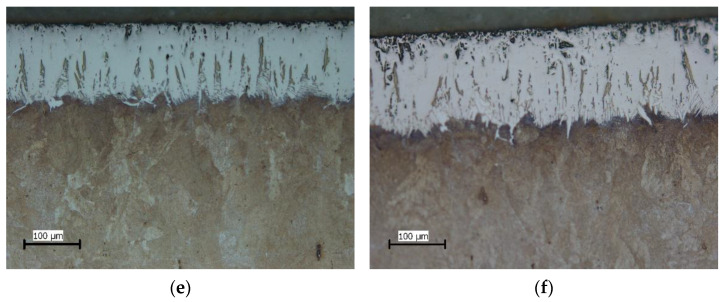
Microstructure of boride layers on C70W2 steel, 200:1 magnification, obtained at: (**a**) 870 °C, 4 h; (**b**) 870 °C, 8 h; (**c**) 920 °C, 4 h; (**d**) 920 °C, 8 h; (**e**) 970 °C, 4 h; (**f**) 970 °C, 8 h.

The microstructure of the layers obtained at different boronizing parameters is shown in Figure 4. It can be observed that with the increase in temperature and/or duration, layers of greater thickness were formed. Porosity is present on the surface of the layer, which is more pronounced at higher boronizing temperatures.

**Figure 5:**
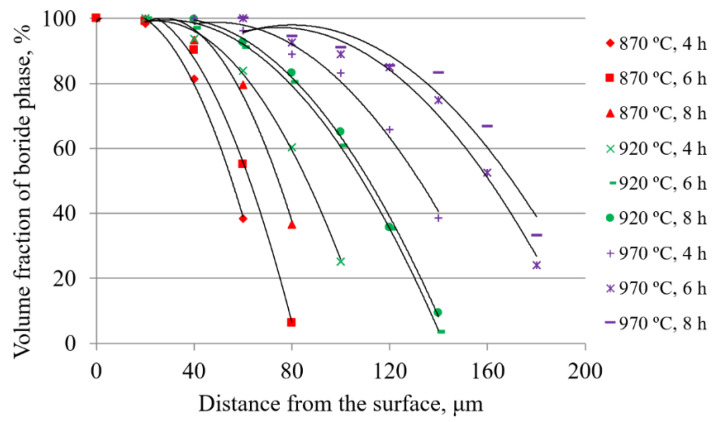
Cross-sectional change in the volume fraction of the boride phase on C70W2 steel.

Analysis of the change in the volume fraction of the boride phase in the cross-section was performed on all samples. The results of the analysis are given in Table 2 and the diagram in Figure 5. The obtained results represent the arithmetic mean of the values obtained by measuring three repetitions for each state of the experimental design.

As can be seen in Table 2 and Figure 5, as the temperature and/or duration of boronizing increases, the volume fractions change in a way that indicates the formation of thicker layers. 

In order to analyze the morphology in more detail, the layer was divided into compact and toothed parts based on the volume fraction of the boride phase. Table 3 shows the results for the thickness of the compact part of the layer, which was determined as described in Section 2. For better analysis, the same table shows the value of the average layer thickness on this steel, obtained in a previous study [30]. These values were determined as described in Section 1, i.e., according to Figure 1. The thickness of the toothed part of the layer, given in Table 3, was calculated as the difference between the average thickness and the thickness of the compact part of the layer

**Figure 6:**
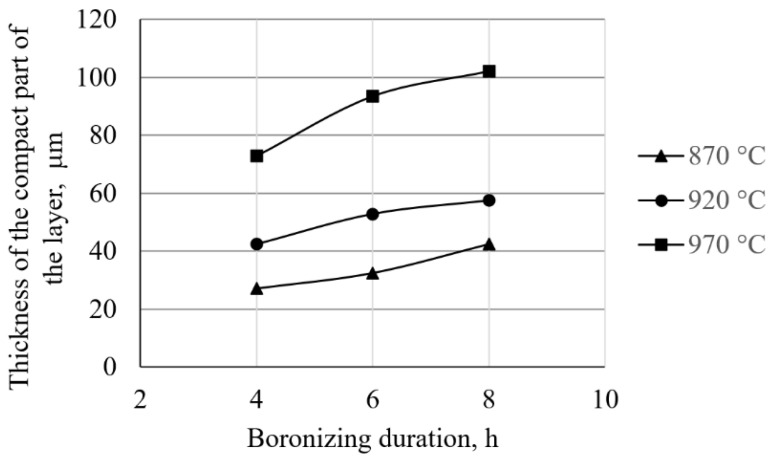
Dependence of the thickness of the compact part of the boride layer on the temperature.

**Figure 7:**
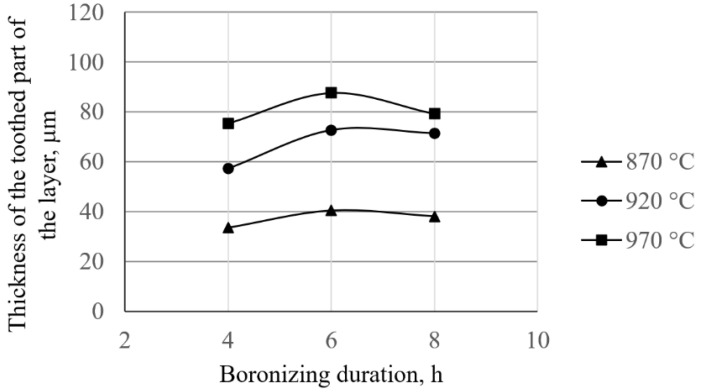
Dependence of the thickness of the toothed part of the boride layer on the temperature.

The dependence of the thickness of the compact and toothed parts of the layer on the temperature and the duration of boronizing is shown in the diagrams in Figure 6 and Figure 7, respectively.

As can be seen in Figure 6, the increase in temperature and/or the duration of boronizing leads to an increase in the thickness of the compact layer. When it comes to the thickness of the toothed part of the layer (Figure 7), it can be seen that boronizing for up to 6 h increases the thickness of the toothed part, and as the boronizing duration increases, the toothness begins to decrease.

As part of the research, the change in cross-sectional microhardness of the layer was analyzed. The hardness was determined as described in Section 2, i.e., according to Equation (1). Previous research found that the temperature and duration of boronizing did not significantly affect the hardness of the Fe_2_B layer. It was also found that the average hardness of the Fe_2_B layer achieved on C70W2 steel is 1557 HV [30]. This value was used as the hardness value of the boride phase in Equation (1). The data given in Table 4 were used as the hardness value of the base material.

The values of the total cross-sectional hardness of the layer, calculated according to Equation (1), are given in Table 5.

### 3.2. Statistical Analysis

Statistical analysis was performed using TIBCO Statistica software [31]. The regression analysis is performed with the results of the volume fraction of the boride phase, the thickness of the compact part of the boride layer, and the thickness of the toothed part of the boride layer. Based on the results, a second-order polynomial regression model was chosen for each analysis. This model contains linear and quadratic terms of the independent variables and their interactions. Analysis of variance (ANOVA) of the regression models was performed to determine which of the terms included in the model were statistically significant. A *p*-value greater than 0.05 supports the null hypothesis of no effect on the output, so terms with *p* > 0.05 were excluded from the regression equation.

To determine if there is a functional relationship between the boronizing parameters (temperature and duration), distance from the surface, and volume fraction of the boride phase, a regression analysis was performed based on the experimental results (Table 2). Analysis of variance (ANOVA) was performed for the second-order polynomial regression model and the results are shown in Table 6.

As can be seen from Table 6, the *p*-values of all included terms are smaller than 0.05, so they are statistically significant. The regression coefficients were calculated using the least-squares method for all terms included in the model, and the regression equation for the prediction of the volume fraction of the boride phase was established as follows:

(2) $$\begin{array}{c} v_{B}=-4058.9+9.15\cdot \vartheta -0.01\cdot \vartheta^{2}+69.77\cdot t-1.40\cdot t^{2}-14.90\cdot x-0.01\cdot x^{2}\\ -0.06\cdot \vartheta \cdot t+0.02\cdot \vartheta \cdot x+0.12\cdot t\cdot x \end{array}$$

where *v_B_* is the volume fraction of boride phase/%, *ϑ* is the boronizing temperature/°C, *t* is the boronizing duration/h, and *x* is the distance from the surface/µm. The adjusted coefficient of determination (Adj *R*^2^) of the obtained regression equation is 0.945950.

Statistical analysis was also performed for the results of the thickness of the compact part and the thickness of the toothed part of the boride layer, which is shown in Table 3. 

The results of ANOVA for the thickness of the compact part of the boride layer are shown in Table 7. ANOVA showed that temperature (linear and quadratic) and duration (linear) have a significant effect on the thickness of the compact part of the boride layer, while their interaction is not significant at the alpha level of 0.05.

A regression analysis was performed to determine whether there is a functional relationship between the boronizing temperature and duration and the thickness of the compact part of the boride layer. ANOVA was performed for the second-order polynomial regression model, and the results are shown in Table 8. The terms with a *p*-value greater than 0.05 are considered statistically insignificant and were therefore excluded from the model.

As can be seen from Table 8, the *p*-values of all included terms are less than 0.05 and therefore statistically significant. The regression coefficients were calculated for all terms included in the model, and the regression equation for the prediction of the thickness of the compact part of the boride layer was established as follows:

(3) $$d_{CB}=3168.223-7.396\cdot \vartheta +0.004\cdot \vartheta^{2}+4.994\cdot t$$

where *d_CB_* is the thickness of the compact part of boride layer/µm, *ϑ* is the boronizing temperature/°C, and *t* is the boronizing duration/h. The adjusted coefficient of determination (Adj *R*^2^) is 0.97201, and it shows that obtained model fits the observations. 

The results of ANOVA for the thickness of the toothed part of the boride layer are shown in Table 9. As can be seen, the influence of temperature (linear and quadratic) and duration (quadratic) on the thickness of the toothed part of the boride layer is significant at the 0.05 alpha level. The linear term of duration has a *p*-value of more than 0.05 (*p* = 0.061159), but this term was included because of the hierarchy.

A regression analysis was performed to determine if there was a functional relationship between the boronizing temperature and duration and the thickness of the toothed part of the boride layer. ANOVA was performed for the second-order polynomial regression model, and the results are shown in Table 10. The terms with a *p*-value greater than 0.05 are considered to be statistically insignificant and were therefore excluded from the model. Although the *p*-value of the linear term of duration is greater than 0.05, this term was included in the model because it is a hierarchical model. 

The regression coefficients were calculated for all terms included in the model, and the regression equation for predicting the thickness of the toothed part of the boride layer was established as follows:

(4) $$d_{TB}=-3482.15+7.10\cdot \vartheta -0.004\cdot \vartheta^{2}+28.16\cdot t-2.16\cdot t^{2}$$

where *d_TB_* is the thickness of the toothed part of the boride layer/µm, *ϑ* is the boronizing temperature/°C, and *t* is the boronizing duration/h. The adjusted coefficient of determination of the obtained regression equation is 0.96958.

**Figure 8:**
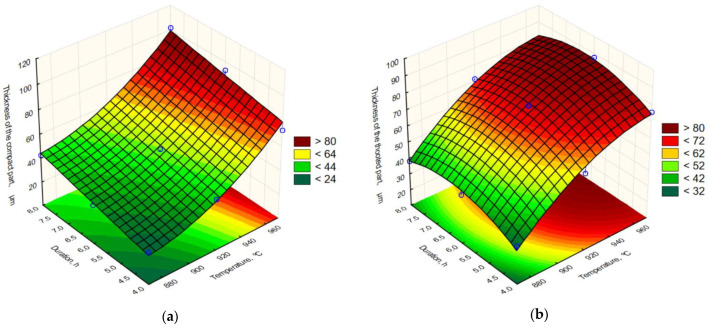
Response surface of regression equation for (**a**) the thickness of the compact part of boride layer; (**b**) the thickness of the toothed part of boride layer.

The graphical representations of Equations (3) and (4) are given in Figure 8a,b, respectively.

## 4. Discussion

### 4.1. Characterization of Boride Layers Obtained on C70W2

Although toothed layers were formed on the surface, it can be concluded from the comparison with the layers formed on C15 and C45 steels boronized in the same medium and with the same parameters [30,32], that this toothing is much less pronounced. This is consistent with research that has shown that both the average thickness and the appearance of the boride layer depend on the material being boronized [33]. Li et al. observed the formation of a compact and relatively smooth boride layer on Cr12Mn2V2 high chromium cast iron as a consequence of the high content of alloying elements [20]. Other authors also confirmed in their studies that boronizing steels with higher carbon content and/or alloying elements results in layers with a smaller thickness and less-pronounced toothness [1,2,3,7,8,34,35]. The reason for this phenomenon is that during the growth of the layer, carbon and alloying elements are pushed under the layer, creating a barrier in the diffusion zone. This barrier hinders the diffusion of boron, resulting in thinner and less-toothed layers. The increased carbide content below the layer, noticeable as a darker area below the boride layer (visible in Figure 4b), is also due to carbon displacement. Non-uniform distribution of carbon in the diffusion zone due to its diffusion, where the carbon content was highest between the boride teeth and lowest at the tip of the teeth, was also noted by Meric et al. [36]. Higher content of carbon in the diffusion zone results in a higher amount of carbide compared to the core. Higher content of carbide in the diffusion zone was confirmed in other studies [2,10,13,34]. Figure 3 and Figure 4 also show the presence of porosity on the surface of the layer, with higher porosity being present at higher boronizing temperatures. This is consistent with observations by Meric et al. who found higher porosity on the samples boronized at higher temperatures [36]. In contrast, Gök et al. found that higher boronizing temperatures resulted in lower layer porosity [37]. Carrera-Espinoza et al. and Jain et al. reported porosity as a characteristic of boride layers obtained on low-carbon steel [3,22]. The porosity of the layer is also reported in other studies [34]. It was generally accepted that the origin of porosity in the boride layer is mainly attributed to the heterogeneous distribution of the boron in the boride layer [22,38].

The data in Table 2 and Figure 5 show a decrease in the share of the boride phase with increasing distance from the surface, which is due to the saw-tooth morphology of the boride layer. If the data for each temperature are compared (Figure 5), it can be observed that with the increase in temperature and/or the duration of boronizing, the boride layer occurs at greater depths, i.e., the layer became thicker. Furthermore, it can be observed that the increase in boronizing temperature had a greater impact on the volume fractions (i.e., the thickness of layers) compared to the duration of boronizing. This dependence of the layer thickness on the duration and temperature of boronizing is consistent with studies dealing with the kinetics of boronizing [1,2,5,7,30,32,35]. These studies showed that increasing the temperature and duration of boronizing results in thicker layers, and it was found that the effect of temperature on the average layer thickness is greater than the effect of the boronizing duration. It can also be seen in Figure 5 that the curves for 6 and 8 h are closer together than the curves for 4 and 6 h, indicating slower diffusion over time. This is also consistent with the boronizing kinetics studies, which confirmed the diffusive nature of boronizing. It was found that boronizing obeys the parabolic law where the square of the layer thickness is proportional to the duration of boronizing, and the proportionality coefficient represents the growth rate constant [1,4,5,7,9,13,24,30,32,39,40]. Since boronizing obeys the parabolic law, this would imply that diffusion slows down over time.

When it comes to the thickness of boride layers, previous research mainly focused on the dependence of the average layer thickness on the process parameters. These studies confirmed the diffusion character of boronizing, and that with the increase in temperature and duration, the average thickness of the boride layer increases, while this growth slows down over time. Unfortunately, this approach to the analysis of boride layers, although adequate when it comes to researching the kinetics of boronizing, does not provide data on the morphology or the dependence of the thickness of the compact and toothed parts on the parameters of boronizing. Given that the behavior of a layer during exploitation depends on its morphology, it can be said that for the complete characterization of layers, it is not sufficient to determine only their average thicknesses. Knowing the change in the volume fraction of the boride phase enables the division of the layer into a compact part (i.e., a part of the layer with a large share of the boride phase) and a toothed part of the layer. This research examined the morphology, and in that sense, the analysis of the influence of boronizing parameters on the thickness of the compact and toothed parts of the layer was performed. When it comes to the compact part of the layer (Table 3, Figure 6), similar behavior can be observed as with the average layer thickness, i.e., with an increasing temperature and/or duration of boronizing, the thickness of the compact part of boride layer increases. The influence of temperature on the thickness of the toothed part is similar to the influence on the thickness of the compact part—higher temperatures resulted in greater thicknesses of the toothed part (Table 3, Figure 7). As for the duration, different behavior can be observed. By boronizing for up to 6 h, the thickness of the toothed part of the layer increases, and with longer boronizing, this value begins to decrease. This would mean that at shorter boronizing durations, the growth of the average layer thickness (i.e., growth “through the teeth”) is faster than the increase in the thickness of the compact part, which results in greater thicknesses of the toothed part. However, with time, the increase in the thickness of the layer (i.e., diffusion “through the teeth”) slows down, and the compact part grows faster, leading to a decrease in the thickness of the toothed part of the layer. A similar but even more pronounced phenomenon was observed in C15 and C45 steels [30,32]. These studies showed that, in both types of steel, the thickness of the toothed part was greatest after 6 h of boronizing, while after 8 h of boronizing, there was a decrease in toothing. It can be noted that this is consistent with the observations of Delai et al. who reported that at a boronizing temperature of 860 °C, increasing the boronizing duration from 4 to 8 h results in very dense and thick boride layers. The same study reported that for a boronizing duration of 8 h, increasing the temperature (from 860 to 900 °C) leads to much thicker and more compact boride layers [40]. This dependence of the thickness of compact and toothed parts on the parameters is interesting because it means that the morphology of the layer changes over time, which is important for the quality of boride layer. In general, thin layers are used for protection against adhesive wear, while thick layers are recommended for abrasive and erosion resistance [11,18,24]. It is to be expected that the compact part of the layer will be the primary carrier of wear resistance during operation. On the other hand, the greater thickness of the compact part does not necessarily imply good wear resistance, since the toothed part is the one that provides the adhesion of the layer to the substrate and ensures that the layer does not peel during operation [3,10].

The experimental results were also statistically analyzed to determine if there was a functional relationship between the boronizing parameters and the observed properties (volume fraction of the boride phase, thickness of the compact part of the layer, and thickness of the toothed part of the layer).

Regression analysis of the volume fraction of the boride phase was performed, and the ANOVA of the selected regression model (Table 6) showed that all independent variables (boronizing temperature, boronizing duration, and distance from the surface), i.e., their linear and quadratic terms, as well as their interactions, have an influence at the 0.05 alpha level of significance. As a result of the regression analysis, Equation (2) was established. The proposed equation allows the prediction of the volume fraction of the boride phase in the observed depth as a function of the boronizing temperature and boronizing duration for C70W2 steel. The value of the adjusted coefficient of determination shows that 94.6% of the total variability of the output data can be explained by the regression equation. A high value of the adjusted coefficient of determination shows that the obtained regression equation fits the empirical data well.

The ANOVA of the thickness of the compact part of the boride layer (Table 7) confirmed the significant influence of both the boronizing temperature and boronizing time, while their interaction was not significant at the 0.05 alpha level. The value of eta squared, sometimes referred to as the percentage contribution, measures the proportion of variation in the dependent variable attributable to each source of variation (independent variables and their interaction) and is often used as a method of measuring the effect size of different variables. The results show that temperature has a stronger effect on the thickness of the compact layer, explaining 87.4% of the total variation. The duration of boronizing explains 10.8% of the total variation in the thickness of the compact part of the boride layer. The regression analysis of the thickness of the compact part showed that the linear and quadratic terms of temperature and the linear term of duration were significant at the alpha level of 0.05. As a result of the regression analysis, Equation (3) was established to predict the thickness of the compact part of the boride layer on C70W2 steel as a function of the boronizing temperature and duration. The value of the adjusted coefficient of determination shows that 97.2% of the total variability of the output data can be explained by the regression equation, so it can be concluded that the obtained regression equation fits the observations.

The thickness of the toothed part of the boride layer was also analyzed using ANOVA and regression analysis. ANOVA has shown that the boronizing temperature and duration have a significant effect on the thickness of the toothed part of the layer. The eta squared values show that the boronizing temperature has a greater contribution to the variation of the thickness of the compact part of the boride layer than the boronizing duration (91.3% vs. 7.2%). Regression analysis was also performed, and the ANOVA for the second-order polynomial regression model showed that the linear term for temperature and the squared term for duration have a *p*-value of less than 0.05, so they are considered statistically significant. Despite the *p*-value of more than 0.05, the linear term for the duration was included in the model because of the hierarchy principle. Furthermore, its regression coefficient is significant at the alpha level of 0.05 (*p* = 0.02462). As a result of the regression analysis, Equation (4) was established. Knowing the boronizing temperature and boronizing duration, this equation allows for the calculation of the thickness of the toothed part of the boride layers on C70W2 steel. The high value of the adjusted coefficient of determination (0.96958) shows that the obtained model agrees with the observations.

The plots in Figure 8a,b represent the response surfaces of Equations (3) and (4), respectively. The surface plot in Figure 8a shows the dependence between the thickness of the compact part of the boride layer and the boronizing temperature and duration. As can be seen, the influence of the temperature is stronger than the influence of the duration, with the greatest thickness being obtained during boronizing with the highest temperature and the longest duration. The response surface in Figure 8b shows the dependence between the thickness of the toothed part of the boride layer and the boronizing temperature and duration. Again, it can be seen that the influence of temperature is greater than the influence of duration. The influences of temperature and duration on the thickness of the toothed part are different. While an increase in temperature causes an increase in the thickness of the toothed part of the layer, an increase in the boronizing duration to approximately 6–6.5 h leads to an increase in the thickness of the toothed part, while further prolongation of the boronizing leads to a decrease in the thickness of the toothed part of the boride layer.

**Figure 9:**
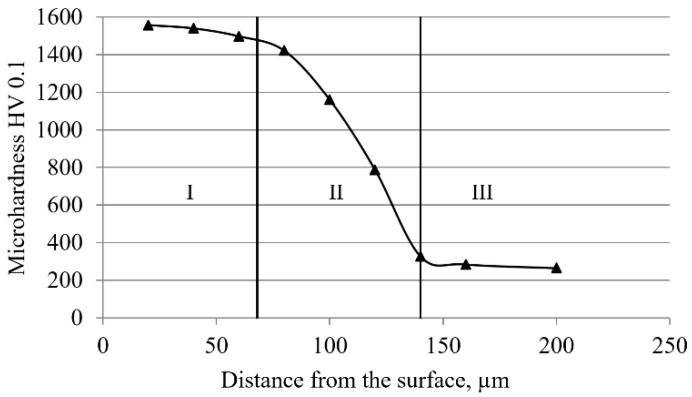
Cross-sectional hardness change of the sample boronized at 920 °C for 6 h.

Regarding hardness, previous studies on the cross-sectional hardness of boride layers determined the hardness as the arithmetic mean of five or more measurements at each depth [2,3,4,5,9,37,39]. This method is suitable for layers parallel to the surface, and it can be said that reliable results are obtained in this case. In the case of the boride layer, its saw-tooth morphology is a problem, as it can lead to significant deviations of the measured hardness from the actual hardness, depending on how many measurements (“passes”) were made “through the tooth” and how many “in between” teeth. Furthermore, due to the morphology of the layer and the very nature of the hardness measurement procedure, it is difficult (or impossible) to measure the hardness of borides in the toothed part of the layer (especially in layers with thinner teeth). Therefore, the measurement of hardness in the diffusion zone is often reduced to the measurement of hardness of the base material between the teeth. Since this hardness is lower than the hardness of boride, this method of measurement can lead to a significant deviation of the results from the actual values. Balusamy et al. pointed out in their research that the total hardness depends not only on the hardness of individual phases, but also on their volume fractions [34], which means that more accurate results can be obtained if the fraction of boride and base material at the individual depths is known. The total hardness is the result of the hardness of individual phases (boride phase and base material) according to their volume fractions at the observed depths. During boronizing, there is a significant increase in hardness on the surface, primarily due to the formation of an extremely hard boride layer, but also due to the increase in the hardness of the substrate in the diffusion zone. As can be seen in Table 4, the hardness of base material in the area between the teeth and directly below the boride layer is higher than the hardness measured at the core, and this value decreases with increasing distance from the surface. This is due to the diffusion of carbon, which is pushed inwards from the surface layers due to the growth of the boride layer. A higher carbon content leads to an increase in the perlite share, i.e., carbide, which results in an increase in the hardness of the base material in the diffusion zone. The values for the total cross-sectional hardness are given in Table 5. As can be seen, the total cross-sectional hardness decreases with increasing distance from the surface. The reason for this is the decreasing share of the boride phase (due to the saw-tooth morphology) and the decreasing hardness of base material in the diffusion zone (which approaches the value of hardness in the core with increasing distance). The surface hardness values for all test specimens are extremely high and range from 1545 to 1557 HV (i.e., correspond to the hardness of Fe_2_B boride). Previous studies have shown that the hardness of iron boride obtained on C70W2 steel is practically independent of the boronizing parameters and ranged between 1488 and 1603 HV, i.e., on average, it was 1557 HV [30]. Figure 9 shows the cross-sectional hardness change curve for C70W2 steel boronized at 920 °C for 6 h.

The appearance of the curve in Figure 9 represents a typical appearance of the hardness curve obtained on the basis of results in Table 5. Based on the hardness, the surface layer can be divided into three distinct areas. Area I is characterized by high hardness and a small decrease in hardness with depth. The high hardness values are due to the large share of the boride phase, and it can be said that this is the area of the compact part of the boride layer. Given the hardness, it is to be expected that, due to the high hardness value and compactness of the layer, the boride layer at these depths will provide the best wear resistance. Area II is the “toothed” part of the layer, characterized by an intense drop in hardness, primarily due to the reduced volume fraction of the boride phase, but also to a decrease in the hardness of the base material in the diffusion zone. In terms of wear resistance, it is to be expected that the material will wear faster in this part of the layer. Area III is the base material. 

### 4.2. Advantages of a New Approach to the Characterization of Boride Layers

Since the objective of this study was not only to investigate the boride layers formed on C70W2 steel, but also to explore the possibility of characterizing boride layers based on the analysis of changes in the volume fraction of the boride phase, this subsection discusses the problems of classical characterization based only on the average thickness of the boride layer, and the advantages of the new approach over the classical one.

In order to discuss the differences in approaches, the data from this study conducted on C70W2 steel and the results of previous research [30,41] conducted on C15 steel were used for the analysis. Furthermore, for the purpose of validation of the method, additional tests were performed on C15 samples, i.e., hardness testing was performed in the cross-section of the layer. The volume fraction of the boride phase on C15 steel is given in Table 11. At the bottom of Table 11, the average layer thickness (*d*_av_) is given for each state of the experimental plan.

As mentioned, additional hardness tests were performed on C15 steel. Table 12 shows the hardness values of the base material every 20 µm from the surface. 

Based on the volume fractions in Table 11 and Equation (1), the cross-sectional hardness of the boride layers on C15 steel was determined. A previous study [30] found that the temperature and duration of boronizing do not significantly affect the hardness of Fe_2_B and that it averages 1541 HV, so this value was used for the calculation according to Equation (1). The results for the cross-sectional hardness of C15 steel are given in Table 13.

**Figure 10:**
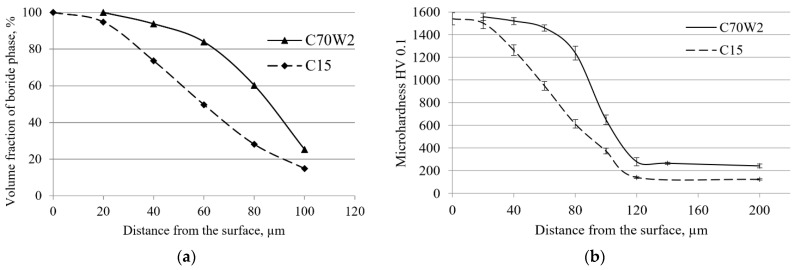
Comparison of the properties of layers of the same average thickness obtained on C70W2 and C15 steels: (**a**) Change in the volume fraction of the boride phase; (**b**) change in cross-sectional hardness.

One of the disadvantages of the classical method of determining the average layer thickness is that it does not indicate anything about the morphology of the layer. Two layers with the same average thickness may be completely different morphologically and during exploitation. Namely, the wider and denser the teeth, the more compact the layer will be, which should be reflected in the hardness values. The analysis based on volume fractions provides the possibility of a more detailed analysis of morphology. To illustrate the advantages of this method, the results for layers of the same thickness obtained on C70W2 and C15 steels are discussed below. If the data from Table 3 and Table 11 are compared, it can be found that the average layer thickness on C70W2 steel, corresponding to state 4 of the experiment design, is approximately equal to the average layer thickness on C15 steel, boronized according to state 2 of the experiment design. The average layer thickness on C70W2 steel boronized at 920 °C for 4 h is 99.7 µm, while the boronizing of C15 steel at 870 °C for 6 h resulted in a layer of approximately the same thickness, i.e., 98.4 µm. The cross-sectional changes in the volume fractions and the hardness curves for these layers are given in Figure 10.

From the graphs in Figure 10, it can be seen that although these layers have approximately the same thickness, their properties are significantly different. As for the volume fraction (Figure 10a), it can be seen that as the distance from the surface increases, the volume fraction of the boride phase decreases in both types of steel, but this decrease is more pronounced in C15 steel. The C70W2 steel retains high shares of the boride phase at greater depths. It can also be seen that the fraction of the boride phase in C70W2 steel is approximately 90% at a depth of approximately 50 µm, while this fraction is present at a depth of approximately 26 µm in C15 steel. The part of the curve where the pronounced decrease in volume fraction occurs corresponds to the toothed part of the layer. It can be observed that in the toothed area, the fraction of the boride phase is significantly higher in C70W2 steel than in C15 steel. The different curves for these layers show differences in their morphology. Although the average thicknesses of both layers are approximately equal, it can be observed that the layer on C70W2 steel has a larger thickness considering the compact part (the part of the layer with a high fraction of the boride phase). Since their average thicknesses are the same, this means less pronounced toothness. When it comes to the toothed part, the diagrams show that these two layers not only have a different thickness of the toothed part, but the toothed part also differs in appearance. The high shares of the boride phase in the toothed part of the C70W2 steel indicate that the boride teeth are thicker and more densely distributed than in the layers obtained on C15 steel. The different morphology of the layers is also reflected in the cross-sectional hardness curves (Figure 10b). The greater thickness of the compact part of the layer on C70W2 steel resulted in high hardness being maintained at greater depths. Higher shares of the boride phase in the toothed part of the layer on C70W2 steel also resulted in higher hardness compared to C15 steel. It should be emphasized that in the case of the boride layer, in addition to the hardness, the degree of toothness also plays an important role in its behavior during use, which guarantees good adhesion of the layer to the substrate.

**Figure 11:**
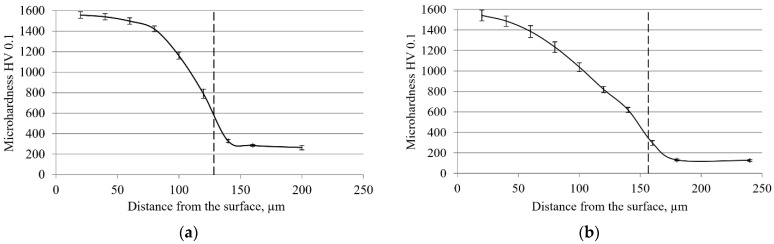
Microhardness curve for layers obtained by boronizing at 920 °C for 6 h: (**a**) C70W2 steel; (**b**) C15 steel.

As mentioned in the Introduction, the term ‘average thickness’ may suggest a false assumption that, at these depths, the hardness values are similar to the hardness of the boride itself. By analyzing the data in Table 2, it can be determined that at depths corresponding to the average layer thickness, the volume fractions of the boride phase on C70W2 steel for the experiment design states 1 to 9 (indicated in Table 3) are as follows: 37, 26, 36, 26, 22, 24, 30, 26, and 38%. Since these are boride layers with less pronounced toothness, this difference should be even more pronounced in low-carbon steels, in which boride layers with a pronounced saw-tooth morphology are formed, characterized by longer, thinner, and more widely spaced teeth. The analysis of data in Table 11 shows that at the average layer thicknesses obtained on C15 steel, the volume fractions of the boride phase are even lower, and the experiment design states 1 to 9 are as follows: 18, 13, 16, 7, 15, 17, 15, 15, and 1%. Such reduced shares should be reflected in the hardness, which, at a depth corresponding to the average thickness of the boride layer, should be significantly lower than the hardness of boride, i.e., the surface hardness. The data in Table 5 show that for C70W2 steel, the hardness values at depths corresponding to the average layer thicknesses for each state of the experiment design are significantly lower than the surface hardness of the boride layer and range from 570 to 840 HV. Similar to the volume fraction, this difference should be even greater for the layers with an extremely pronounced saw-tooth morphology. To confirm this assumption, the cross-sectional hardness of the layers obtained on C15 steel was determined based on the volume fractions and Equation (1), and the results are shown in Table 13. A comparison of the average layer thickness with the data in Table 13 shows that the hardness at depths corresponding to the average thickness of the boride layer on C15 steel ranges from 180 to 455 HV. Figure 11 shows the change in cross-sectional hardness of C70W2 and C15 steels boronized at a temperature of 920 °C for 6 h. The depths corresponding to the average layer thickness are marked with a dashed line in the diagrams.

As can be seen in Figure 11, the average thicknesses are in a range where the hardness is significantly lower than the surface hardness. This is particularly pronounced in C15 steel, where the average thickness is practically in zone III, i.e., the zone corresponding to the base material. All these data suggest that, when it comes to hardness and practical application of boronizing, data on the average layer thickness should be taken with caution because the hardness at these depths is significantly lower than the surface hardness. The more pronounced the toothness of the layer, the greater the difference in hardness. Thus, for layers with extremely pronounced toothness, the hardness at the depth corresponding to the average thickness is only slightly higher than the hardness of the core. 

In view of the above, it can be said that the analysis based on the volume fractions is a simple method that allows more accurate characterization of the boride layers and offers many advantages over the classical method. When it comes to layer thickness, it can be defined based on the depth at which the adequate fraction of the boride phase is located. Furthermore, the curve of the change of the volume fraction not only provides insight into the morphological character of the layer, but the layer can also be divided into compact and toothed parts. This method of characterization opens new possibilities for research in the field of tribology. In previous studies, tribological investigations focused on analyzing the behavior of boride layers using different test methods and under different wear conditions [27,33,36]. Although the results of these studies were quantitative, their relationship with the layer thickness and the type of material was qualitative. The division of the layer into compact and toothed parts would enable further research in order to quantitatively relate the morphology of the boride layer with its tribological properties, i.e., to investigate the influence of the thickness of the compact part of the layer and its toothed part on the layer behavior under different wear conditions.

An additional advantage of the analysis based on volume fractions is the more accurate determination of cross-sectional hardness, which is of great importance in industrial applications since certain machine parts have requirements not only for surface hardness, but also for hardness at a certain depth. More accurate data on the cross-sectional hardness distribution opens the possibility of defining the effective boride layer thickness based on the hardness value that provides effective wear resistance. This approach to determining the average thickness of thermodiffusion layers is not uncommon, as the thicknesses of cemented and nitrided layers are also determined on the basis of hardness.

## 5. Conclusions

In this research, the boride layers were tested on C70W2 steel using a new approach to characterization based on the variation in the volume fraction of the boride phase (Fe_2_B) in the cross-section of the layer. According to the obtained results, the following conclusions can be summarized:

- The metallographic analysis showed that boride layers with less-pronounced toothness were formed on the surface of the C70W2 steel than is the case with low-carbon steels.
- Analysis of the volume fraction of the boride phase in the cross-section of the sample showed that with increasing temperature and duration, the boride phase was found at greater depths, i.e., thicker layers were formed. It was also found that the effect of boronizing temperature on the volume fractions is greater than the effect of duration. The volume fraction curves confirmed the diffusion character of boronizing, i.e., the diffusion of boron slows down over time.
- Based on the volume fractions, the layers are divided into compact and toothed parts. The study showed that the boronizing parameters affect the layer morphology. Higher temperatures resulted in greater thicknesses of the compact and toothed parts. On the other hand, it was found that the duration of boronizing has a different effect on the morphology of the layer compared to the temperature. Increasing the boronizing duration resulted in greater thicknesses of the compact part, while the toothness first increased and then began to decrease. The maximum thickness of the toothed part was reached by boronizing for 6 h. This phenomenon means that thicker but less-toothed layers are formed with longer boronizing.
- Based on the volume fraction and the hardness of the boride phase and the base material, the cross-sectional hardness was determined. It was found that the hardness of the base material in the diffusion zone is higher compared to the core due to the higher amount of carbon directly below the boride layer. The highest hardness (approximately 1554 HV) was achieved on the sample surface due to the high share of the boride phase. As the distance from the surface increases, the hardness decreases, primarily due to the lower share of the boride phase, but also due to the decreasing hardness of the base material, which approaches the hardness values in the core as the distance from the surface increases.
- At the distance corresponding to the average thickness of the boride layer, there is 22–38% of the boride phase, and the achieved hardness at these depths is 570–840 HV. The difference between the surface hardness and hardness at the depth corresponding to the average layer thickness is even greater in layers with more pronounced saw-tooth morphology.
- Based on the surface hardness, it is possible to divide the layer into three areas. Area I is the area with extremely high hardness (due to the large share of boride phase), and this area corresponds to the compact part of the layer. Area II is the area where there is a significant drop in hardness (primarily due to the lower share of boride phase), and this area corresponds to the toothed part of the layer. Area III is the area of the base material.
- The statistical analysis showed that there is a functional relationship between the boronizing parameters and the observed properties of the boride layer (volume fraction of boride phase, thickness of the compact part of the layer, and thickness of the toothed part of the layer). The results showed that the boronizing temperature has a stronger effect on the observed properties than the boronizing duration.
- As a result of the regression analysis, three regression equations (for the prediction of the volume fraction of the boride phase at the considered depth, the thickness of the compact part, and the thickness of the toothed part of the boride layer on C70W2 steel as a function of the boronizing temperature and duration) were estimated.

Regarding this new approach to the characterization of layers, it can be concluded that the characterization of boride layers based on volume fractions offers multiple advantages over the conventional methods:

- The effective layer thickness can be determined based on the depth at which the desired share of boride phase is obtained.
- Based on the volume fraction of the boride phase, it is possible to divide the layer into the compact and toothed parts, and on this basis, monitor the influence of the parameters on the layer morphology.
- It is possible to accurately determine the changes in the cross-sectional hardness, which opens the possibility of defining the effective layer thickness as the depth at which the desired hardness is achieved.

## Figures and Tables

**Table 1:** The chemical composition of C70W2 steel, wt. %.

Steel	C	Si	Mn	P	S	Cu
C70W2	0.72	0.211	0.286	0.014	0.017	-

**Table 2:** Volume fraction of the boride phase for steel C70W2, %.

Distance from the Surface, µm	*ϑ* = 870 °C			*ϑ* = 920 °C			*ϑ* = 970 °C		
	*t* = 4 h	*t* = 6 h	*t* = 8 h	*t* = 4 h	*t* = 6 h	*t* = 8 h	*t* = 4 h	*t* = 6 h	*t* = 8 h
0	100.0	100	100.0	100.0	100.0	100.0	100.0	100.0	100.0
20	98.4	99.2	99.9	100.0	100.0	100.0	100.0	100.0	100.0
40	81.3	90.2	93.3	93.8	97.1	99.6	100.0	100.0	100.0
60	38.4	55.1	79.5	84.0	91.2	92.6	96.2	100.0	100.0
80	0	6.3	36.6	60.3	80.5	83.2	88.9	92.7	94.5
100		0	2.1	25.2	60.9	65.1	83.2	88.9	91.0
120			0	0	35.3	35.7	65.7	85.1	85.3
140					3.5	6.3	38.6	74.9	83.2
160					0	0	1.9	52.5	66.7
180							0	24.0	33.1
200								0.8	2.9
220								0	0

**Table 3:** Thicknesses of the compact and toothed parts of the boride layers on C70W2 steel, μm.

Boronizing Temperature, °C	Boronizing Duration, h	Experiment Design State	Thickness of the Compact Part of the Layer, μm	Average Boride Layer Thickness, μm [30]	Thickness of the Toothed Part of the Layer, μm
870	4	1	27	61 ± 9	34
	6	2	32	73 ± 9	40
	8	3	43	81 ± 10	38
920	4	4	42	100 ± 8	57
	6	5	53	129 ± 9	76
	8	6	58	129 ± 11	71
970	4	7	73	148 ± 9	75
	6	8	93	181 ± 12	88
	8	9	102	181 ± 10	79

**Table 4:** Cross-sectional microhardness of the transition zone on boronized C70W2 steel, HV 0.1.

Distance from the Surface, µm	*ϑ* = 870 °C			*ϑ* = 920 °C			*ϑ* = 970 °C		
	*t* = 4 h	*t* = 6 h	*t* = 8 h	*t* = 4 h	*t* = 6 h	*t* = 8 h	*t* = 4 h	*t* = 6 h	*t* = 8 h
20	946								
40	858	904	1094	956	968				
60	421	620	897	941	882	938	922		
80	279	312	501	753	857	819	829	944	933
100	272	285	293	343	546	469	764	833	863
120	254	283	277	278	364	321	591	736	812
140		265	271	266	283	285	339	678	808
160			268	285	285	263	273	513	644
180							267	335	397
200							255	290	300
Core	254	250	241	242	265	235	248	253	254

**Table 5:** Cross-sectional microhardness of the boride layer on C70W2 steel, HV 0.1.

Distance from the Surface, µm	*ϑ* = 870 °C			*ϑ* = 920 °C			*ϑ* = 970 °C		
	*t* = 4 h	*t* = 6 h	*t* = 8 h	*t* = 4 h	*t* = 6 h	*t* = 8 h	*t* = 4 h	*t* = 6 h	*t* = 8 h
20	1547	1545	1557	1557	1557				
40	1427	1494	1526	1520	1540	1552	1557		
60	857	1136	1422	1459	1498	1512	1533	1557	1557
80	279	391	888	1238	1421	1433	1477	1513	1512
100	272	285	319	649	1161	1178	1424	1477	1481
120		283	277	278	788	762	1226	1435	1446
140		265	271	266	328	365	809	1337	1369
160			268		285	263	298	1061	1123
180							267	628	676
200								290	300
Core	254	250	241	242	265	235	248	253	254

**Table 6:** Analysis of variance for the regression model of the volume fraction of the boride phase.

Effect	Sum of Squares SS	Degrees of Freedom df	Mean Square MS	*F*-Value	*p*-Value
Intercept	1180.448	1	1180.448	20.277	0.000046
(1) Temperature	1238.853	1	1238.853	21.281	0.000032
Temperature^2^	1250.232	1	1250.232	21.476	0.000030
(2) Duration	696.147	1	696.147	11.958	0.001183
Duration^2^	374.437	1	374.437	6.432	0.014669
(3) Distance from the surface	8599.073	1	8599.073	147.711	0.000000
Distance from the surface^2^	5605.214	1	5605.214	96.284	0.000000
(1) × (2)	520.616	1	520.616	8.943	0.004463
(1) × (3)	7643.142	1	7643.142	131.291	0.000000
(2) × (3)	2045.926	1	2045.926	35.144	0.000000
Error	2677.912	46	58.215		

**Table 7:** Analysis of variance of the thickness of the compact part of the boride layer.

Factor	Sum of Squares SS	Degrees of Freedom df	Mean Square MS	*F*-Value	*p*-Value	Eta Squared
(1) Temperature L + Q	4840.170	2	2420.085	124.9680	0.000054	0.874
(2) Duration L	598.494	1	598.494	30.9049	0.002590	0.108
Error	96.828	5	19.366			0.017
Total	5535.491	8				

**Table 8:** Analysis of variance for the regression model of the thickness of the compact part of the boride layer.

Effect	Sum of Squares SS	Degrees of Freedom df	Mean Square MS	*F*-Value	*p*-Value
Intercept	175.655	1	175.6551	9.0705	0.029697
(1) Temperature	201.919	1	201.9190	10.42667	0.023231
Temperature^2^	233.369	1	233.3692	12.0507	0.017822
(3) Duration	598.494	1	598.4937	30.9049	0.002590
Error	96.828	5			

**Table 9:** Analysis of variance of the thickness of the toothed part of the boride layer.

	Sum of Squares SS	Degrees of Freedom df	Mean Square MS	*F*-Value	*p*-Value	Eta Squared
(1) Temperature L + Q	2989.536	2	1494.768	120.0107	0.000269	0.913
(2) Duration L + Q	236.729	2	118.365	9.5032	0.030229	0.072
Error	49.821	4	12.455			0.015
Total	3276.087	8				

**Table 10:** Analysis of variance for the regression model of the thickness of the toothed part of the boride layer.

Effect	Sum of Squares SS	Degrees of Freedom df	Mean Square MS	*F*-Value	*p*-Value
Intercept	212.061	1	212.061	17.0258	0.014538
(1) Temperature	186.121	1	186.121	14.9431	0.018060
Temperature^2^	164.105	1	164.105	13.1755	0.022164
(3) Duration	174.550	1	174.550	14.0142	0.020059
Duration^2^	153.654	1	153.654	12.3364	0.024620
Error	49.821	4	12.455		

**Table 11:** Volume fraction of the boride phase for steel C15, %.

Distance from the Surface, µm	*ϑ* = 870 °C			*ϑ* = 920 °C			*ϑ* = 970 °C		
	*t* = 4 h	*t* = 6 h	*t* = 8 h	*t* = 4 h	*t* = 6 h	*t* = 8 h	*t* = 4 h	*t* = 6 h	*t* = 8 h
0	100.0	100.0	100.0	100.0	100.0	100.0	100.0	100.0	100.0
20	87.2	94.7	95.0	95.9	100.0	100.0	100.0	100.0	100.0
40	55.9	73.6	77.0	83.1	92.0	96.4	97.9	100.0	100.0
60	28.2	49.6	56.8	64.1	81.9	81.8	85.9	96.0	99.1
80	5.5	28.1	38.0	44.6	70.3	72.5	73.8	87.3	91.1
100	0	14.8	19.9	21.3	56.9	60.4	63.5	76.8	80.4
120		0	0	9.3	44.2	46.0	53.0	74.6	76.4
140				0	31.1	34.3	36.6	65.2	67.9
160					9.9	16.0	21.9	50.3	57.9
180					0	0	4.6	34.3	42.9
200							0	18.5	27.9
220								5.7	15.2
240								0	3.6
260									0
*d*_av_, µm	70	98	104	118	157	158	173	211	239

**Table 12:** Cross-sectional microhardness of the transition zone on boronized C15 steel, HV 0.1.

Distance from the Surface, µm	*ϑ* = 870 °C			*ϑ* = 920 °C			*ϑ* = 970 °C		
	*t* = 4 h	*t* = 6 h	*t* = 8 h	*t* = 4 h	*t* = 6 h	*t* = 8 h	*t* = 4 h	*t* = 6 h	*t* = 8 h
20	764	824	873	876					
40	448	490	690	721	856	935	946		
60	204	364	442	496	680	820	625	925	863
80	160	250	216	321	500	488	495	699	728
100	143	171	150	172	371	300	331	625	626
120	136	140	132		246	239	257	476	459
140		140			202	184	207	422	391
160		131			159	129	155	294	273
180							132	238	242
200								187	196
220									155

**Table 13:** Cross-sectional microhardness of the boride layer on C15 steel, HV 0.1.

Distance from the Surface, µm	*ϑ* = 870 °C			*ϑ* = 920 °C			*ϑ* = 970 °C		
	*t* = 4 h	*t* = 6 h	*t* = 8 h	*t* = 4 h	*t* = 6 h	*t* = 8 h	*t* = 4 h	*t* = 6 h	*t* = 8 h
0	1541	1541	1541	1541					
20	1441	1503	1508	1513	1541	1541	1541		
40	1059	1263	1345	1402	1486	1519	1528	1541	1541
60	581	948	1067	1165	1384	1410	1412	1516	1535
80	236	613	719	865	1232	1252	1266	1434	1468
100	143	374	427	463	1037	1049	1099	1329	1362
120	136	140	132	254	819	838	938	1271	1285
140					619	649	695	1151	1172
160					296	355	459	921	1007
180					130	129	197	685	799
200								437	571
220								211	366
240									177
Core	128	123	126	123	126	125	126	129	126

## Data Availability

Not applicable.
